# Exploring Stressors and Coping Strategies of Young Care Workers in Long‐Term Care: A Qualitative Study Using the CHEERS Framework

**DOI:** 10.1002/nop2.70839

**Published:** 2026-10-21

**Authors:** Chia‐Chen Chang, Chen‐Yin Tung

**Affiliations:** ^1^ Department of Health Promotion and Health Education, College of Education National Taiwan Normal University Taipei Taiwan

**Keywords:** CHEERS framework, coping strategies, long‐term care, psychological resilience, stress, young care workers

## Abstract

**Aims:**

To explore the psychological stressors and coping strategies of young care workers aged 30 years or younger across diverse care settings.

**Design:**

This qualitative study was conducted using data collected through focus group interviews to investigate stress experiences and coping responses.

**Methods:**

Four focus group interviews were conducted with a purposive sample of 16 young care workers. Data were analysed using grounded theory methods involving open, axial and selective coding.

**Results:**

Young care workers encounter psychological stressors from boundary violations, including physical acts like client violence and emotional pressures from excessive demands, which impair their well‐being and caregiving effectiveness. A coping framework emerged, comprising six strategies—compassion, help‐seeking, empathy, exposure, reflection and sensitivity—structured across three stages: primary coping, secondary coping, and long‐term coping to alleviate stress and enhance resilience across varied settings.

**Conclusion:**

Findings highlight the significant stressors faced by young care workers and a structured coping framework that supports their resilience and occupational well‐being.

**Implications for Nursing Practice:**

The CHEERS framework may inform training and supervision strategies for young care workers and support nurse educators and long‐term care nursing leaders in strengthening workforce retention and care quality.

**Impact:**

This study tackled the pervasive stress among young care workers, revealing a framework of coping strategies to mitigate its effects. It impacts care facilities and workers by offering tools to improve resilience and care quality.

**Reporting Method:**

This study adhered to the COREQ criteria.

**Patient or Public Contribution:**

Young care workers participated in the focus group interviews and shared their experiences of occupational stress and coping. Immediate member checking was conducted at the end of each focus group to allow participants to clarify or confirm the researchers' summary of the discussion. Participants were not involved in manuscript preparation.

**Abbreviations**
CCS: community care settings
CHEERS: Compassion, Help‐seeking, Empathy, Exposure, Reflection and Sensitivity
ICS: institutional care settings

## 1 Introduction

### 1.1 Care Workers and Occupational Stress in Long‐Term Care

Care workers play a crucial role in long‐term care (LTC), providing physical assistance with daily activities as well as emotional and social support to older adults, particularly those with dementia or disabilities (Huang et al. 2025). Their work involves substantial physical and emotional demands that may adversely affect their well‐being (Huang et al. 2025). In addition to providing direct care, care workers must communicate and coordinate with clients' family members, which may add to the emotional demands of their work (Janssen and Abbott 2023; Kane et al. 2023). Common challenges faced by direct care workers include time pressure, understaffing and the emotional impact of caregiving, as well as difficulties in interacting with older people's families (Jones et al. 2021; Huang et al. 2025).

These stressors are associated with poorer well‐being and increased risk of burnout among long‐term care and healthcare assistants (Jones et al. 2021; Chamberlain et al. 2016; Huang et al. 2025). Care workers in long‐term care settings may encounter substantial workloads, emotional demands and challenges in establishing professional boundaries (Huang et al. 2025; Miller et al. 2023; Norful et al. 2024). Resilience may facilitate psychological adaptation to occupational stress (Kunzler et al. 2022; Cooper et al. 2021; Norful et al. 2024), while workforce retention is also shaped by interpersonal and organisational factors within long‐term care settings (Miller et al. 2023). Supporting care workers may therefore contribute to both staff well‐being and the quality and continuity of care for older adults.

### 1.2 Emotional Strain and Psychological Adaptation Among Young Care Workers

Care workers may experience substantial emotional strain as they adapt to caregiving demands, with the nature of these demands varying across care settings (Huang et al. 2025; Scales and Lepore 2020). Understanding these stressors is important for developing appropriate emotional and organisational support strategies, particularly because family, colleague and supervisory support may buffer adverse mental health outcomes among long‐term care workers (Liu et al. 2025; Janssen and Abbott 2023). Professional boundary management is particularly relevant in home care contexts, where workers may encounter requests beyond their defined responsibilities (Karlsson et al. 2020).

Institutional settings may provide more formalised work structures and access to supervisory and team‐based resources, although organisational support varies across settings (Berta et al. 2022; Norful et al. 2024). However, the intensity of work demands and hierarchical management structures may introduce additional psychological stress (Molero Jurado et al. 2018; Krein et al. 2022). Challenges in maintaining professional boundaries may add to the emotional demands of caregiving, particularly in settings where close relationships with clients develop over time (Hulen et al. 2022; Lam and Baxter 2023).

By contrast, community‐based and home care settings present distinct relational and organisational challenges. The personalised nature of care may blur professional boundaries as workers develop close relationships with clients and families. Home care workers may also work in relative isolation, with limited opportunities for immediate peer support and shared workplace interaction (Lam and Baxter 2023; Leverton et al. 2021; Karlsson et al. 2020). Additionally, the independent nature of community‐based care may limit immediate peer support, further complicating stress management and professional adaptation (Gion and Abitz 2019).

Regardless of the care setting, young care workers must develop effective emotional regulation strategies and establish appropriate professional boundaries to mitigate the long‐term impact of stress on their mental health. Implementing tailored support mechanisms specific to each care environment can enhance emotional resilience, reduce burnout and improve workforce retention while maintaining high‐quality care standards.

### 1.3 Coping Strategies and the Need for a Structured Framework

Developing strong coping strategies is essential for managing workplace stress and fostering psychological resilience in caregiving roles (Navarro Prados et al. 2022; Cooper et al. 2021). Resilience refers to the capacity to adapt to occupational adversity and maintain functioning under sustained workplace demands (Foster et al. 2018; Cooper et al. 2021; Kunzler et al. 2022). Prior research has documented that excessive job stress in healthcare is linked to burnout and diminished well‐being, underscoring the importance of effective coping mechanisms to safeguard mental health (Janssen and Abbott 2023).

Long‐term care workers exposed to high job demands and insufficient workplace support may be at greater risk of poorer psychological well‐being, burnout and turnover (Huang et al. 2025; Janssen and Abbott 2023). Conversely, organisational and social support may contribute to better psychological well‐being and lower turnover intention among long‐term care workers (Liu et al. 2025). Resilience may further support workers' capacity to adapt to occupational adversity and maintain psychological well‐being in demanding care environments (Cooper et al. 2021).

Similarly, in LTC settings, frontline workers benefit from strategies that help them process emotional stress, such as peer support, counselling or skills training in self‐care (Gion and Abitz 2019; Odle‐James and Willie‐Tyndale 2023). While many studies on health professionals have identified key stressors and coping methods, there is comparatively limited research focusing specifically on the coping strategies of young care workers in LTC contexts. This gap highlights the need for a clearer understanding of how young care workers handle stress and what interventions might bolster their psychological adaptation to the caregiving role.

To address these issues, the present study investigates the following key questions:

1. What are the main sources of occupational stress for young care workers in institutional vs. community‐based LTC settings?
2. How do young care workers cope with these stressors, and what specific strategies or resources do they employ?
3. How can these coping strategies be organised into a comprehensive framework to enhance young care workers' resilience, mental health and workplace adaptation?

Through qualitative analysis, this study explores the lived experiences of young care workers, examining how they manage work‐related stress and how these coping practices contribute to their psychological well‐being and professional growth. By identifying effective coping strategies unique to this cohort, the research aims to inform targeted training programmes and workplace interventions. Ultimately, supporting young caregivers in stress management and resilience‐building can improve their mental health and job adjustment, thereby promoting workforce stability and elevating the quality of long‐term care services for older adults.

## 2 Method

This qualitative study employed focus group interviews guided by grounded theory principles to explore the work‐related stressors and coping strategies of young care workers in long‐term care settings. Grounded theory was chosen for its iterative, inductive approach, allowing themes to emerge directly from participants' narratives rather than pre‐existing hypotheses (Corbin and Strauss 1990). The study adhered to the Consolidated Criteria for Reporting Qualitative Research (COREQ) guidelines to ensure comprehensive reporting (see Supplementary File S1) (Tong et al. 2007).

### 2.1 Study Setting and Recruitment

The study was conducted in Taiwan across diverse care environments, including one residential long‐term care facility (institutional care setting, ICS) and three community‐based units (two home‐care agencies and one day‐care centre; community care settings, CCS). Recruitment leveraged professional networks affiliated with St. John University and was facilitated by facility managers who identified staff meeting the inclusion criteria. A total of 20 potential candidates were initially recruited. However, four candidates were excluded because they were older than 30 years. All 16 eligible care workers agreeded to participate in the study.

### 2.2 Sample

The final sample comprised 16 care workers (7 females, 9 males) with a mean age of  25.44 years (SD = 2.48) and mean work experience of 1.96 years (SD = 1.60). To facilitate in‐depth interaction, participants were divided into four focus groups with sizes of 5, 5, 2 and 4 participants respectively. The variance in group size, particularly the smallest group (*n* = 2), reflected the strict enforcement of eligibility criteria, as originally scheduled candidates for that session were excluded due to age limits.

Inclusion criteria were: (1) graduation from a university programme in long‐term care, (2) aged ≤ 30 years and (3) current employment in ICS or CCS. Regarding exclusion criteria, participants with cognitive impairments or those with less than 6 months of experience were excluded. The latter criterion was established to minimise the confounding effects of initial ‘reality shock’ (Kramer 1974). We aimed to recruit care workers who had moved beyond the initial survival phase and had begun to develop stable coping patterns and reflective capacities, which are essential for constructing the conceptual framework.

Ultimately, the sample was balanced with eight participants from ICS and eight from CCS (see Table 1 for demographics). To minimise selection bias, recruitment emphasised diversity in gender, workplace type and geographic location within Taiwan. However, towards the end of recruitment, we noted an underrepresentation of participants with less than 1 year of experience, despite additional outreach efforts. This limitation is further addressed in Section 4.

**TABLE 1:** Basic information on focus group interview participants.

No.	Gender	Age (years)	Years of service	Setting
A	Male	26	2.0	ICS
B	Female	25	3.7	ICS
C	Male	30	7.2	ICS
D	Male	23	1.0	CCS
E	Female	23	1.4	ICS
F	Female	26	1.0	CCS
G	Female	22	2.0	CCS
H	Female	22	1.0	CCS
I	Female	29	2.0	CCS
J	Male	26	1.0	CCS
K	Female	25	0.5	CCS
L	Male	30	1.3	CCS
M	Male	25	0.9	ICS
N	Male	25	2.1	ICS
O	Male	25	2.2	ICS
P	Male	25	2.0	ICS

Abbreviations: CCS, community care setting; ICS, institutional care setting.

### 2.3 Data Collection

Data were collected through four focus group interviews conducted between March and July 2023. A semi‐structured interview guide was developed based on previous research on occupational stress, emotional well‐being, professional boundaries and coping among long‐term care and home care workers (Smith‐MacDonald et al. 2019; Janssen and Abbott 2023; Karlsson et al. 2020) and validated through expert review by two qualitative researchers and a long‐term care practitioner. The guide covered four domains: (1) background and interactions with clients and colleagues, (2) types of work‐related stressors, (3) emotional responses and coping strategies and (4) future expectations. Open‐ended questions (e.g., ‘What challenges do you face in your daily work?’) were paired with probes to elicit detailed responses. The guide was piloted with two care workers, confirming its clarity and relevance, with no revisions needed.

The interviews were conducted by the lead researcher, a female PhD‐prepared registered nurse and associate professor with over a decade of experience in qualitative research. This expertise ensured effective facilitation and attentiveness to group dynamics. Participants, who were unacquainted with the research team, were briefed on the study and provided written informed consent. Sessions were conducted in private meeting rooms at the respective participating institutions to ensure convenience and comfort. Interviews lasted 1.5–2 h (mean = 1.8 h) and were audio‐recorded with permission.

To ensure data collection rigour, an assistant moderator used a standardised Focus Group Interview Checklist (see Supplementary File S2) throughout all sessions. The checklist was used to document group dynamics and non‐verbal cues, such as collective nodding, silence and agitation, to provide contextual information for the interview data. At the conclusion of each session, the moderator summarised the main points discussed and invited participants to clarify or confirm the summary as a form of immediate member checking. Data saturation was reached after four sessions, when no new concepts emerged in the final group. Participants received USD 50 (NTD 2000) as compensation.

### 2.4 Data Analysis

Data were analysed using grounded theory methods, involving open, axial and selective coding (Corbin and Strauss 1990). Audio recordings were transcribed verbatim within 1 week by a professional transcriptionist, with transcripts cross‐checked for accuracy by a research team of three medical education researchers with qualitative expertise.

The analysis proceeded iteratively. First, open coding involved a line‐by‐line review to identify initial concepts (e.g., ‘ignoring the client’ or ‘venting to friends’). Second, axial coding was used to link these concepts into broader categories (e.g., ‘Boundary Setting’ or ‘Social Support’). Finally, selective coding refined these categories into a core explanatory framework—the CHEERS framework—to describe how young care workers balance stress and resilience. Table 2 illustrates this analytical trajectory, providing a clear visual audit trail from raw data to the final themes.

**TABLE 2:** Coding tree illustrating the analytical process: From raw participant quotes to the CHEERS Framework.

Raw data	Open coding	Axial coding	Selective coding
(Participant quotes)	(In vivo codes)	(Sub‐categories)	(CHEERS strategy)
‘My mood improves a bit… [understanding it is] dementia‐related’ (FG4‐206‐15)	Perspective taking/Shared suffering	Empathy & Altruism (Transforming affect)	Empathy (Primary Coping)
‘He used his functional hand to touch me… I temporarily left the scene to ensure safety’ (FG4‐202‐16)	Physical withdrawal/Ensuring safety	Boundary Setting (Behavioural adaptation)	Exposure (Primary Coping)
‘Our supervisor's encouragement keeps us motivated… I turn to my friends to vent’ (FG1‐22‐18)	Venting emotions/Seeking supervision	Social Support (External resources)	Help‐seeking (Secondary Coping)
‘It's heartbreaking for both the family and patient… I try to stand in their shoes’ (FG3‐124‐20)	Understanding pathology/Not taking it personally	Cognitive Reframing (Internal regulation)	Compassion (Secondary Coping)
‘I write it down… thinking about why it happened and what I can do better’ (FG1‐28‐21)	Journaling/Self‐dialogue	Cognitive Processing (Post‐event analysis)	Reflection (Long‐term Coping)
‘They began to yell loudly… I noticed the signs of sundowning immediately’ (FG4‐147‐7)	Detecting behavioural cues/Anticipation	Professional Sensitivity (Proactive observation)	Sensitivity (Long‐term Coping)

To ensure analytical rigour, the research team held biweekly analysis meetings. Any coding discrepancies were resolved through consensus discussions. During these sessions, the team revisited the original audio recordings and the Focus Group Interview Checklist to clarify the context and intent of participants' statements, thereby ensuring that the final themes were robust and credible.

### 2.5 Ethical Considerations

This study adhered to the Declaration of Helsinki and was approved by the Research Ethics Committee of National Taiwan Normal University (Ref. No. 202106ES003). Participants were informed of the study aims, procedures and their right to withdraw at any time without penalty. Written informed consent was obtained from all participants, including consent for audio recording. To ensure confidentiality, all personal identifiers were removed, and the data were stored on a secure, password‐protected server accessible only to the research team.

### 2.6 Rigour and Reflexivity

Rigour was maintained throughout data collection and analysis by addressing credibility and confirmability, consistent with established criteria for trustworthiness in qualitative research (Lincoln and Guba 1985). Credibility was supported by cross‐referencing interview transcripts with observational field notes collected using the Focus Group Interview Checklist. In addition, immediate member checking was conducted at the conclusion of each focus group, during which participants were invited to clarify or confirm the moderator's summary of the discussion. Confirmability was supported through biweekly research team meetings, during which coding decisions and interpretations were reviewed and discussed with reference to the original transcripts and field notes. An audit trail was maintained to document the development of codes, categories and the CHEERS framework, with illustrative examples presented in Table 2.

Reflexivity and Positionality were critical to the analytical process. The lead researcher, an experienced long‐term care educator, initially recognised a tendency to view participants' narratives through a ‘clinical competency lens’—subconsciously evaluating whether young workers' actions adhered to standard protocols. For instance, early in the analysis, a participant's choice to ‘ignore’ an aggressive client was initially critiqued as professional negligence or avoidance. To mitigate this bias, the team engaged in reflexive dialogues, consciously shifting the analytical focus to a ‘strengths perspective’. This pivot allowed the researchers to reframe these behaviours not as failures, but as adaptive coping mechanisms. Consequently, behaviours initially coded as ‘distraction’ were reinterpreted as Sensitivity (environmental scanning), and ‘ignoring’ was identified as Exposure (strategic boundary setting). These insights were foundational to the development of the CHEERS framework.

## 3 Results

Through four focus group interviews with 16 young care workers aged ≤ 30 years, the analysis identified key occupational stressors and coping strategies across institutional and community‐based long‐term care settings.

### 3.1 Boundary‐Violating Stressors

Stressors primarily arise from boundary violations—physical (e.g., client violence) and emotional (e.g., excessive demands)—disrupting care workers' well‐being. Physical breaches, such as a bedridden client touching intimate areas (‘He used his functional hand to touch me’, FG4‐202‐16), prompted immediate withdrawal to ensure safety (‘Temporarily leave the scene’, FG4‐147‐20).

Emotional violations occurred when clients rejected advice or demanded off‐duty tasks (e.g., ‘You might as well clean the toilet’, FG2‐81‐2), straining mutual trust and emotional boundaries. Persistent violations risked boundary collapse, impairing caregiving efficacy. Effective boundary management thus emerges as critical for protecting care workers' rights and enhancing care quality.

### 3.2 Coping Capacities: The CHEERS Framework

This study identified six coping strategies employed by young care workers to mitigate occupational stressors. These strategies were systematically categorised within the Care Worker CHEERS Framework (Compassion, Help‐seeking, Empathy, Exposure, Reflection, Sensitivity). The CHEERS framework was derived from the data through the coding process illustrated in Table 2. The interpretation of participants' experiences was supported by immediate member checking conducted at the end of each focus group. As depicted in Figure 1, the framework organises the coping responses identified across institutional and community‐based long‐term care settings and provides a conceptual basis for understanding psychological adaptation and caregiving responses.

- Empathy: Understanding clients' conditions (e.g., dementia‐related refusal) reduced personal distress (e.g., ‘My mood improves a bit’, FG4‐206‐15).
- Exposure: Hands‐on adaptation to unreasonable demands involved explaining constraints (e.g., ‘We actually can't do this, grandma’, FG2‐82‐15), fostering stable care relationships.
- Compassion: Care workers transformed negative emotions into empathy, bolstering confidence (‘I try to stand in their shoes’, FG3‐124‐20).
- Help‐seeking: Professional (supervision) and social (colleagues, friends) support alleviated overwhelm (e.g., ‘Our supervisor's encouragement keeps us motivated’, FG1‐26‐03).
- Sensitivity: Observing client cues (e.g., sundowning agitation) enabled proactive responses (‘They began to yell loudly’, FG4‐147‐7).
- Reflection: Journaling or discussions post‐event enhanced resilience (e.g., ‘I write it down… it's just this problem’, FG1‐28‐21).

**FIGURE 1:**
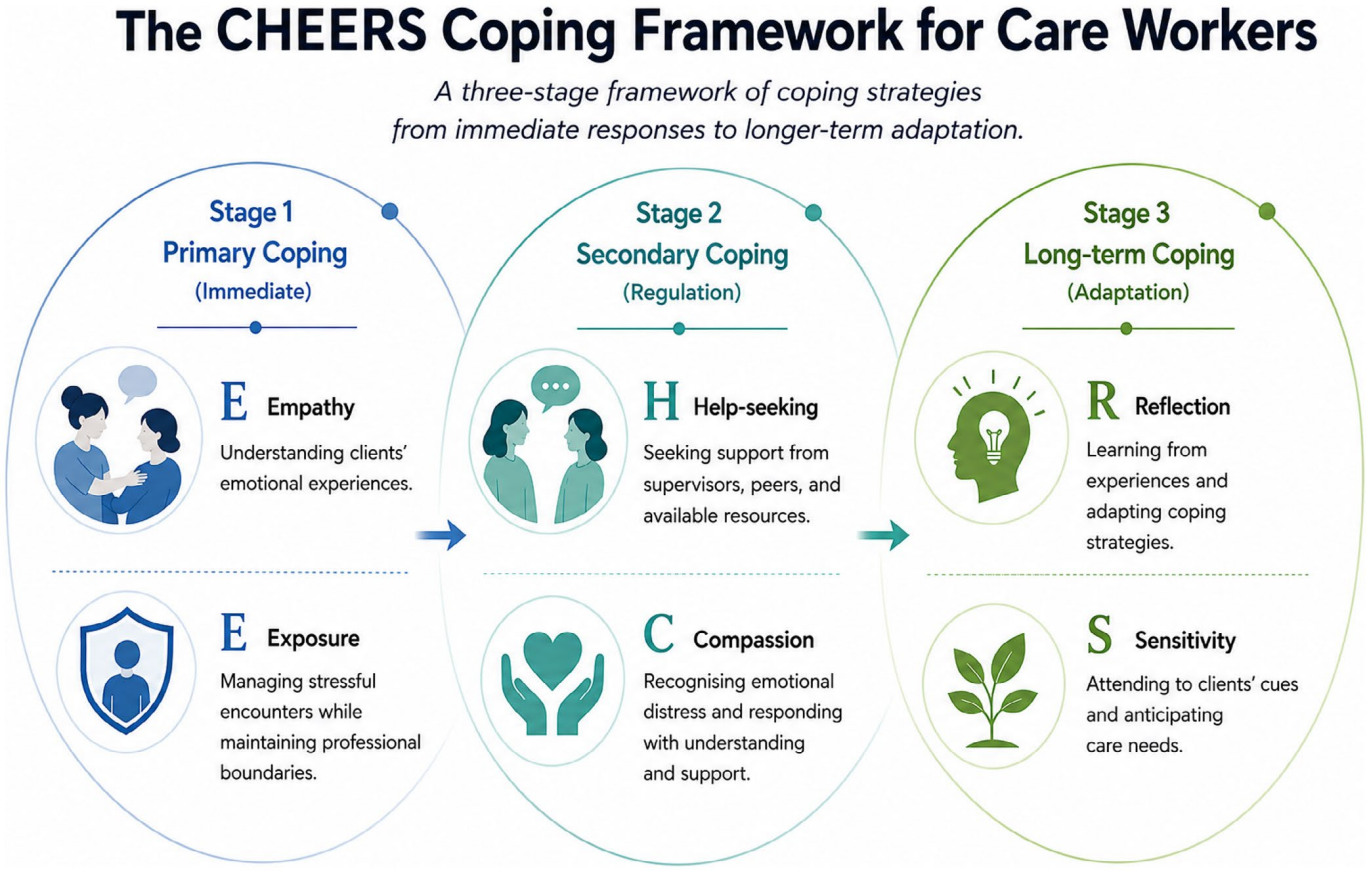
The care worker CHEERS framework: A three‐stage model of coping strategies. This figure illustrates the progression of coping strategies across three stages: (1) Primary Coping (Immediate): Strategies used in the moment of stress (Empathy, Exposure); (2) Secondary Coping (Regulation): Strategies used for emotional processing and support (Help‐seeking, Compassion); (3) Long‐term Coping (Resilience): Strategies used for sustained adaptation and prevention (Reflection, Sensitivity). See Table 2 for the coding audit trail mapping participant quotes to these specific strategies. This figure was created with the assistance of an AI‐based graphic generation tool, and all conceptual content and structure were defined by the authors.

### 3.3 Coping Across Settings

Young care workers in long‐term care settings dedicate substantial time to assisting clients with physical care, psychological support and daily tasks while managing age‐ or illness‐related changes. Through four focus group interviews (*N* = 16) in institutional and community‐based settings, this study identified stressors and coping strategies, organised into three stages within the CHEERS framework: Primary, Secondary and Long‐term Coping.

#### 3.3.1 Stage 1: Primary Coping—Immediate Responses to Stressors

Primary coping strategies address immediate stressors, particularly boundary violations disrupting physical and emotional well‐being. Physical breaches, such as a client's violent behaviour (‘He was shouting, throwing objects’, FG4‐155‐18) or inappropriate touching (‘He used his functional hand to touch me’, FG4‐202‐16), prompted swift actions like withdrawal (‘Temporarily leave the scene’, FG4‐147‐20). Emotional violations arose from excessive demands (e.g., ‘You might as well clean the toilet’, FG2‐81‐2), straining off‐duty boundaries.

- Empathy: Care workers' understanding of clients' circumstances (e.g., dementia‐related refusal) helped reduce their own emotional burden (‘My mood improves a bit’, FG4‐206‐15; ‘I won't attach too much importance’, FG2‐54‐17).
- Exposure: Hands‐on adaptation involved explaining limits (e.g., ‘We actually can't do this, grandma’, FG2‐82‐15), fostering stable care relationships despite unreasonable requests.

These strategies mitigated acute stress and preserved caregiving efficacy in the moment.

#### 3.3.2 Stage 2: Secondary Coping—Support and Emotional Regulation

Secondary coping emerged when immediate responses proved insufficient, particularly under persistent boundary violations or emotional toil. Prolonged demands (e.g., ‘She kept talking for 25 to 30 min’, FG4‐202‐5) or rejection of advice undermined trust, risking boundary collapse.

- Help‐seeking: Care workers sought professional support from supervisors (‘Supervisor's encouragement keeps us motivated’, FG1‐26‐208) or colleagues (‘Experienced colleagues helped me out’, FG4‐147–8), alongside emotional solace from friends (‘I turn to my friends’, FG1‐22‐18). Training courses also bolstered skills (e.g., ‘Dementia course…caregiving skills’, FG1‐32‐22).
- Compassion: Care workers recognised the emotional distress experienced by clients and their families and responded with supportive actions, enabling them to transform emotional strain into more stable patterns of interaction (e.g., ‘It's heartbreaking for both the family and patient’, FG3‐124‐20).

These strategies provided external resources and emotional recalibration to manage ongoing strain.

#### 3.3.3 Stage 3: Long‐Term Coping—Resilience and Proactive Adaptation

Long‐term coping reflected efforts to sustain adaptation and manage recurring occupational stress. Care workers described reflective and anticipatory capacities that supported ongoing adaptation to recurring challenges.

- Reflection: Post‐event journaling or discussions enhanced emotional management (e.g., ‘I write it down… it's just this problem’, FG1‐28‐21; ‘How do I feel about my emotions?’, FG4‐182‐15), fostering resilience for future challenges.
- Sensitivity: Observing client cues (e.g., sundowning agitation: ‘They began to yell loudly’, FG4‐147‐7) enabled proactive responses like calming techniques, reducing escalation risks.

## 4 Discussion

This study explored the stressors and coping strategies of young care workers across diverse caregiving environments. The following discussion engages with existing literature to analyse how coping strategies address specific stressors and examines their application within a broader contextual framework, aligning the study's findings with ongoing academic discourse.

The primary coping stage focuses on care workers' rapid responses to immediate stressors, particularly physical and emotional boundary violations. Empathy aids in understanding clients' needs and moderating personal distress, consistent with research on its role in caregiving resilience (Teófilo et al. 2019; Wu 2021). Additionally, the Exposure strategy, particularly communicating service limitations and withdrawing from potentially harmful interactions, is consistent with research on boundary management among home care aides (Hulen et al. 2022; Lam and Baxter 2023; Karlsson et al. 2020). These primary coping mechanisms demonstrate potential for immediate protection of care workers' well‐being, resonating with studies on short‐term stress adaptation.

The secondary coping stage addresses persistent stressors, particularly when boundary violations threaten emotional stability. Compassion emerged as a means through which participants reframed emotionally difficult encounters. This finding can be considered alongside research highlighting the importance of compassion satisfaction and the emotional consequences of compassion fatigue in nursing practice (Xie et al. 2021). This aligns with findings that direct client interactions may evoke empathetic responses, reinforcing care quality (Smith‐MacDonald et al. 2019). Help‐seeking emerges as a key strategy, leveraging supervisory guidance and peer support to mitigate strain, reflecting the importance of organizational resources in the literature (Janssen and Abbott 2023; Liu et al. 2025).

The long‐term coping stage reflects care workers' efforts to sustain adaptation in the face of recurring occupational stress. Reflection may facilitate the processing of work‐related experiences, strengthen professional insight and support ongoing adaptation. Previous studies have highlighted the relevance of reflective practice to professional learning and reflective ability, while workplace reflection‐based interventions have shown potential to support nurses' resilience (Nishimoto et al. 2023; Patel and Metersky 2022; Muir et al. 2022). Sensitivity, characterised by attention to changes in clients' behaviours and contextual cues, may enable care workers to recognise emerging needs and respond appropriately. Research on person‐centred dementia care similarly emphasises the importance of recognising behavioural and interactional cues, identifying residents' underlying needs and adapting care accordingly (Sefcik et al. 2020; Kim et al. 2024). Together, Reflection and Sensitivity may support longer‐term adaptive caregiving, although their effects on burnout require further longitudinal investigation.

Variations in coping preferences among care workers highlight the influence of workplace dynamics on adaptive responses (Krein et al. 2022; Molero Jurado et al. 2018). Rather than reflecting distinct environmental dichotomies, these differences suggest a spectrum of strategies shaped by contextual factors such as client interactions and organisational support (Janssen and Abbott 2023; Leverton et al. 2021; Karlsson et al. 2020). This contextual variation suggests that training and support strategies may need to account for differences in relational demands, professional boundaries and access to organisational support across long‐term care settings.

## 5 Conclusion

This study elucidates the stressors encountered by young care workers and their coping strategies, framed within the novel Care Worker CHEERS Framework, across diverse long‐term care settings. Boundary‐violating stressors, such as physical violations (e.g., client violence) and emotional demands (e.g., excessive requests), significantly challenge care workers' well‐being and efficacy. Through a three‐stage coping process—primary, secondary and long‐term—participants employed strategies including compassion, help‐seeking and reflection to foster resilience. Variations in coping preferences underscore the influence of workplace environments, reflecting a spectrum of adaptive responses from structured support to direct engagement. These findings align with existing research on stress adaptation and boundary management, highlighting the critical role of flexible coping mechanisms in sustaining care quality and worker health, and offering a fresh framework to enhance caregiver support across contexts.

## 6 Limitation

The findings of this study should be interpreted in light of several limitations. First, although data saturation was achieved, the sample size of 16 participants from Taiwan limits the transferability of the findings to broader populations or different cultural contexts. Cultural and organisational contexts may influence how the coping strategies identified in this study are expressed, and their applicability to other settings requires further investigation. Second, the use of focus groups may have introduced social desirability bias. While this method effectively captured group consensus, participants might have been less likely to disclose sensitive interpersonal conflicts with supervisors compared to individual interviews. Third, participants with less than 6 months of experience were excluded to focus on established coping strategies rather than the acute stress of initial adaptation. Future research could investigate how novices acquire these strategies during their transition period.

## 7 Implications for Nursing Practice

The CHEERS framework provides practical implications for the education, supervision and support of young care workers in long‐term care settings. The findings highlight the importance of addressing not only empathy and relational skills but also assertiveness and professional boundary setting. Nurse educators may incorporate the Exposure strategy into simulation‐based training to help learners practise appropriate responses to aggression and unreasonable demands while maintaining professional boundaries. Nurse managers and long‐term care nursing leaders may also apply the Help‐seeking strategy by strengthening supervisory guidance and peer support, particularly for community‐based care workers who may have limited access to immediate workplace support. These approaches may support young care workers' coping and well‐being while contributing to workforce retention and care quality. Future longitudinal research should examine how the CHEERS framework can be applied in education and supervision and whether reflection‐based interventions support adaptive coping and well‐being over time.

## Author Contributions

**Chia‐Chen Chang:** methodology, data curation, formal analysis, funding acquisition, visualization, project administration, resources, writing – original draft, writing – review and editing, validation. **Chen‐Yin Tung:** conceptualization, supervision, writing – review and editing, visualization, validation.

## Funding

This study was funded by the Ministry of Science and Technology, Taiwan (MOST 112‐2410‐H‐003‐176).

## Conflicts of Interest

The authors declare no conflicts of interest.

## Supporting information


**File S1:** Consolidated Criteria for Reporting Qualitative Research (COREQ) checklist.

**File S2:** Focus Group Interview Checklist & Field Notes.

## Data Availability

The focus group interview data that support the findings of this study are available from the corresponding author upon reasonable request.

## References

[nop270839-bib-0001] Berta, W. B. , C. Stewart , and A. Baumann . 2022. “What Is Old Is New Again: Global Issues Influencing Workers and Their Work in Long‐Term Care.” Healthcare Policy 17: 66–90. 10.12927/hcpol.2022.26853 PMC9346581 35848557

[nop270839-bib-0002] Chamberlain, S. A. , M. Hoben , J. E. Squires , and C. A. Estabrooks . 2016. “Individual and Organizational Predictors of Health Care Aide Job Satisfaction in Long Term Care.” BMC Health Services Research 16: 577. 10.1186/s12913-016-1815-6 PMC5064796 27737672

[nop270839-bib-0003] Cooper, A. L. , J. A. Brown , and G. D. Leslie . 2021. “Nurse Resilience for Clinical Practice: An Integrative Review.” Journal of Advanced Nursing 77: 2623–2640. 10.1111/jan.14763 33559262

[nop270839-bib-0004] Corbin, J. M. , and A. Strauss . 1990. “Grounded Theory Research: Procedures, Canons, and Evaluative Criteria.” Qualitative Sociology 13: 3–21.

[nop270839-bib-0005] Foster, K. , C. Cuzzillo , and T. Furness . 2018. “Strengthening Mental Health Nurses' Resilience Through a Workplace Resilience Programme: A Qualitative Inquiry.” Journal of Psychiatric and Mental Health Nursing 25: 338–348. 10.1111/jpm.12467 29920873

[nop270839-bib-0006] Gion, T. , and T. Abitz . 2019. “An Approach to Recruitment and Retention of Certified Nursing Assistants Using Innovation and Collaboration.” JONA: The Journal of Nursing Administration 49: 354–358. 10.1097/NNA.0000000000000767 31335518

[nop270839-bib-0007] Huang, S. , S. Y. Yau , Y. K. L. Lee , J. Song , Y. Guo , and D. Dong . 2025. “Job Characteristics, Personal Characteristics and Well‐Being of Nursing Assistants in Long‐Term Care Facilities: A Mixed Methods Systematic Review and Narrative Synthesis.” International Journal of Nursing Studies 161: 104934. 10.1016/j.ijnurstu.2024.104934 39476575

[nop270839-bib-0008] Hulen, E. , M. Lafferty , A. Laliberte , S. Saha , and S. T. Edwards . 2022. “Balancing the Benefits of Patient‐Clinician Relationships With Professional Boundaries in Home‐Based Primary Care.” Home Health Care Services Quarterly 41: 330–340. 10.1080/01621424.2022.2056106 35348032

[nop270839-bib-0009] Janssen, L. M. , and K. M. Abbott . 2023. “‘It Hits Me Right Here at My Heart’: Promoting Emotional Health of Home Care Workers.” Journal of Applied Gerontology 42: 680–688. 10.1177/07334648221127690 36305376

[nop270839-bib-0010] Jones, M. T. , R. A. Heckenberg , B. J. Wright , and S. Hodgkin . 2021. “Understanding the Nature and Impact of Occupational Stress on Australian Rural Aged Care Workers.” Health & Social Care in the Community 29: 643–653. 10.1111/hsc.13338 33704853

[nop270839-bib-0011] Kane, L. , C. Leighton , H. Limbrick , S. Kilinc , J. Ling , and J. Eberhardt . 2023. “You Clapped, You Cheered, but Did Anybody Hear? A Mixed‐Methods Systematic Review of Dementia Homecare Workers' Training and Psychosocial Needs.” Home Health Care Services Quarterly 42: 282–310. 10.1080/01621424.2023.2246415 37585717

[nop270839-bib-0012] Karlsson, N. D. , P. K. Markkanen , D. Kriebel , C. J. Galligan , and M. M. Quinn . 2020. “‘That's Not My Job’: A Mixed Methods Study of Challenging Client Behaviors, Boundaries, and Home Care Aide Occupational Safety and Health.” American Journal of Industrial Medicine 63: 368–378. 10.1002/ajim.23082 31833084

[nop270839-bib-0013] Kim, D. , Y. R. Choi , Y. N. Lee , and S. O. Chang . 2024. “Nurses' Shared Subjectivity on Person‐Centered Care for Behavioral and Psychological Symptoms of Dementia in Nursing Homes.” Journal of Nursing Research 32: e330. 10.1097/jnr.0000000000000611 38727209

[nop270839-bib-0014] Kramer, M. 1974. Reality Shock: Why Nurses Leave Nursing. Mosby Yearbook Inc.

[nop270839-bib-0015] Krein, S. L. , M. Turnwald , B. Anderson , and D. T. Maust . 2022. “‘Sometimes It's Not About the Money… It's the Way You Treat People…’: A Qualitative Study of Nursing Home Staff Turnover.” Journal of the American Medical Directors Association 23: 1178–1184. 10.1016/j.jamda.2021.11.036 34990586

[nop270839-bib-0016] Kunzler, A. M. , A. Chmitorz , N. Röthke , et al. 2022. “Interventions to Foster Resilience in Nursing Staff: A Systematic Review and Meta‐Analyses of Pre‐Pandemic Evidence.” International Journal of Nursing Studies 134: 104312. 10.1016/j.ijnurstu.2022.104312 35853312

[nop270839-bib-0017] Lam, J. , and J. Baxter . 2023. “‘Friend‐Ish’: Home Care Workers, ‘Social Labor’ and Managing the Boundaries of the Carer Relationship.” Journal of Applied Gerontology 42: 581–588. 10.1177/07334648221144026 36476078

[nop270839-bib-0018] Leverton, M. , A. Burton , J. Beresford‐Dent , et al. 2021. “‘You Can't Just Put Somebody in a Situation With no Armour’. An Ethnographic Exploration of the Training and Support Needs of Homecare Workers Caring for People Living With Dementia.” Dementia (London) 20: 2982–3005. 10.1177/14713012211023676 PMC8678657 34111969

[nop270839-bib-0019] Lincoln, Y. S. , and E. G. Guba . 1985. Naturalistic Inquiry. Sage.

[nop270839-bib-0020] Liu, Y. L. , L. C. Pien , G. H. Liu , et al. 2025. “Dual Caregiving and Mental Health Among Long‐Term Care Nursing Assistants: The Moderating Role of Social Support.” Journal of Advanced Nursing 81: 3036–3044. 10.1111/jan.16456 39278664

[nop270839-bib-0021] Miller, V. J. , L. Maziarz , J. Wagner , J. Bell , and M. Burek . 2023. “Nursing Assistant Turnover in Nursing Homes: A Scoping Review of the Literature.” Geriatric Nursing 51: 360–368. 10.1016/j.gerinurse.2023.03.027 37104907

[nop270839-bib-0022] Molero Jurado, M. D. M. , M. D. C. Pérez‐Fuentes , J. J. G. Gázquez Linares , M. D. M. Simón Márquez , and Á. Martos Martínez . 2018. “Burnout Risk and Protection Factors in Certified Nursing Aides.” International Journal of Environmental Research and Public Health 15: 1116. 10.3390/ijerph15061116 PMC6025175 29848982

[nop270839-bib-0023] Muir, K. J. , J. Webb‐Jones , N. Farish , K. Barker , C. Miller‐Davis , and S. Galloway . 2022. “‘Room to Reflect’: A Pilot Workplace Resiliency Intervention for Nurses.” International Journal of Environmental Research and Public Health 19: 7272. 10.3390/ijerph19127272 PMC9224273 35742534

[nop270839-bib-0024] Navarro Prados, A. B. , S. Jiménez García‐Tizón , and J. C. Meléndez . 2022. “Sense of Coherence and Burnout in Nursing Home Workers During the COVID‐19 Pandemic in Spain.” Health & Social Care in the Community 30: 244–252. 10.1111/hsc.13397 PMC8250978 33894094

[nop270839-bib-0025] Nishimoto, A. , M. Fukada , R. Okuda , and C. Tanimura . 2023. “Career Stage‐Specific Predictors of Reflective Ability Among Clinical Nurses.” Journal of Advanced Nursing 79: 3981–3996. 10.1111/jan.15710 37212517

[nop270839-bib-0026] Norful, A. A. , K. C. Brewer , K. M. Cahir , and A. M. Dierkes . 2024. “Individual and Organizational Factors Influencing Well‐Being and Burnout Amongst Healthcare Assistants: A Systematic Review.” Int J Nurs Stud Adv 6: 100187. 10.1016/j.ijnsa.2024.100187 PMC11080559 38746791

[nop270839-bib-0027] Odle‐James, T. , and D. Willie‐Tyndale . 2023. “Companions' Chronicles: Experiences of Home Care Workers in the Community Elder Care (Companion) Program in Barbados.” Journal of Applied Gerontology 42: 589–596. 10.1177/07334648221143611 36484408

[nop270839-bib-0028] Patel, K. M. , and K. Metersky . 2022. “Reflective Practice in Nursing: A Concept Analysis.” International Journal of Nursing Knowledge 33: 180–187. 10.1111/2047-3095.12350 34626459

[nop270839-bib-0029] Scales, K. , and M. J. Lepore . 2020. “Always Essential: Valuing Direct Care Workers in Long‐Term Care.” Public Policy & Aging Report: praa022. 10.1093/ppar/praa022. PMC7499724 38626233

[nop270839-bib-0030] Sefcik, J. S. , C. Madrigal , A. R. Heid , et al. 2020. “Person‐Centered Care Plans for Nursing Home Residents With Behavioral and Psychological Symptoms of Dementia.” Journal of Gerontological Nursing 46: 17–27. 10.3928/00989134-20201012-03 PMC8274316 33095889

[nop270839-bib-0031] Smith‐MacDonald, L. , L. Venturato , P. Hunter , et al. 2019. “Perspectives and Experiences of Compassion in Long‐Term Care Facilities Within Canada: A Qualitative Study of Patients, Family Members and Health Care Providers.” BMC Geriatrics 19: 128. 10.1186/s12877-019-1135-x PMC6503362 31060500

[nop270839-bib-0032] Teófilo, T. J. S. , R. F. S. Veras , V. A. Silva , N. M. Cunha , J. D. S. Oliveira , and S. C. Vasconcelos . 2019. “Empathy in the Nurse‐Patient Relationship in Geriatric Care: An Integrative Review.” Nursing Ethics 26: 1585–1600. 10.1177/0969733018787228 30071772

[nop270839-bib-0033] Tong, A. , P. Sainsbury , and J. Craig . 2007. “Consolidated Criteria for Reporting Qualitative Research (COREQ): A 32‐Item Checklist for Interviews and Focus Groups.” International Journal for Quality in Health Care 19: 349–357. 10.1093/intqhc/mzm042 17872937

[nop270839-bib-0034] Wu, Y. 2021. “Empathy in Nurse‐Patient Interaction: A Conversation Analysis.” BMC Nursing 20: 18. 10.1186/s12912-021-00535-0 PMC7802140 33435957

[nop270839-bib-0035] Xie, W. , L. Chen , F. Feng , et al. 2021. “The Prevalence of Compassion Satisfaction and Compassion Fatigue Among Nurses: A Systematic Review and Meta‐Analysis.” International Journal of Nursing Studies 120: 103973. 10.1016/j.ijnurstu.2021.103973 34102372

