# Supplementary material for: Exploring Stressors and Coping Strategies of Young Care Workers in Long‐Term Care: A Qualitative Study Using the CHEERS Framework
Source: Nurs Open. 2026 Oct 21;13(10):e70839. doi: 10.1002/nop2.70839 (PMC13687581; doi:10.1002/nop2.70839)
Supplement: Supplementary file 2 [file NOP2-13-e70839-s002.pdf]

Supplementary File S2
Focus Group Interview Checklist & Field Notes

Focus Group Interview Checklist & Field Notes

Study Title: *Exploring Stressors and Coping Strategies of Young Care Workers*
Date: __________________ **Time:** Start: _______ End: _______
Location: __________________ (e.g., Meeting Room A, [Institution Name])
Moderator: __________________ **Observer:** __________________
Session ID: (e.g., FG1) **Number of Participants:** _______

Part 1: Pre-Session Preparation

- **[ ] Written informed consent was obtained from all participants.**
- **[ ] Audio recording devices (×2) were tested and activated.**
- **[ ] Seating arrangement was recorded (see Seating Chart below).**
- **[ ] Refreshments were provided.**

Part 2: Seating Chart

(Please draw a simple diagram of the seating arrangement to track speakers.)

Part 3: Observational Field Notes

(Record non-verbal cues, emotions and overall atmosphere.)

Topic / Theme	Key Observations & Non-verbal Cues	Observer's Reflective Notes
1. Opening / Ice-breaking	Notes: (e.g., P3 remained silent initially; the moderator used humour to ease the group into discussion.)	
2. Work Stressors *(e.g., Client Aggression)*	Notes: (e.g., When P4 mentioned “being hit,” several participants looked up; the atmosphere became noticeably tense.)	
3. Coping Strategies *(e.g., Ignoring / Running away)*	Notes: (e.g., When P2 said “I got injured,” one participant gasped; others shook their heads to indicate avoidance.)	
4. Organizational Support *(e.g., Supervisors)*	Notes: (e.g., Participants nodded in agreement when discussing support from home care supervisors.)	

Part 4: Immediate Member Checking

- **[ ] Summary: The moderator verbally summarised the key themes that emerged (e.g., need for boundaries, lack of support).**
- **[ ] Confirmation: Did participants agree with the summary?**
  - **□ Yes, full agreement**
  - **□ Partial agreement (participant corrections/additions: ________________)**
- **[ ] Closing: Participants were thanked and compensation was provided (NTD 2,000).**
